# Development of *Leptolyngbya* sp. BL0902 into a model organism for synthetic biological research in filamentous cyanobacteria

**DOI:** 10.3389/fmicb.2024.1409771

**Published:** 2024-07-22

**Authors:** Hong Gao, Yali Wang, Ziling Huang, Feiqi Yu, Xi Hu, Degang Ning, Xudong Xu

**Affiliations:** ^1^Institute of Hydrobiology, Chinese Academy of Sciences, Wuhan, China; ^2^School of Life Sciences, Central China Normal University, Wuhan, China

**Keywords:** synthetic biology, model organism, γ-linolenic acid, *patX-hetR*, filamentous cyanobacteria

## Abstract

Cyanobacteria have great potential in CO_2_-based bio-manufacturing and synthetic biological studies. The filamentous cyanobacterium, *Leptolyngbya* sp. strain BL0902, is comparable to *Arthrospira* (*Spirulina*) *platensis* in commercial-scale cultivation while proving to be more genetically tractable. Here, we report the analyses of the whole genome sequence, gene inactivation/overexpression in the chromosome and deletion of non-essential chromosomal regions in this strain. The genetic manipulations were performed via homologous double recombination using either an antibiotic resistance marker or the CRISPR/Cpf1 editing system for positive selection. A *desD*-overexpressing strain produced γ-linolenic acid in an open raceway photobioreactor with the productivity of 0.36 g·m^−2^·d^−1^. Deletion mutants of predicted *patX* and *hetR*, two genes with opposite effects on cell differentiation in heterocyst-forming species, were used to demonstrate an analysis of the relationship between regulatory genes in the non-heterocystous species. Furthermore, a 50.8-kb chromosomal region was successfully deleted in BL0902 with the Cpf1 system. These results supported that BL0902 can be developed into a stable photosynthetic cell factory for synthesizing high value-added products, or used as a model strain for investigating the functions of genes that are unique to filamentous cyanobacteria, and could be systematically modified into a genome-streamlined chassis for synthetic biological purposes.

## 1 Introduction

The goal of synthetic biology is to build biological systems that are able to perform the desired functions for various purposes, such as producing chemicals (Ro et al., 2006) or biofuels (d’Espaux et al., 2015), detecting trace contaminants (Webster et al., 2014), creating new disease models or treatment methods (Ruder et al., 2011; Hutmacher et al., 2015), or studying basic science issues (Davies, 2017). To this end, synthetic biology adopts a bottom up approach to build biological systems with the ideal characteristics or to reconstruct the existing natural systems by learning from the engineering principles of design and construction (Mukherji and van Oudenaarden, 2009). The engineered biological systems are based on the availability of standardized and characterized bioparts, from which biodevices and then complex biosystems are assembled. This would also need a host and a set of models for a predictable outcome (Mukherji and van Oudenaarden, 2009). Therefore, synthetic biology is implemented by making genetic alterations in the host cells, while the host serves as a platform for achieving design goals using various tools and strategies. The basic requirements for a host include rapid and robust growth, complete genome sequence and gene annotation, facile genetic engineering and in-depth research in physiology and genetics (Kim et al., 2017). Initial studies in synthetic biology depended on model heterotrophic organisms such as *Escherichia coli* and *Saccharomyces cerevisiae*. With the constant extension of application fields of synthetic biology, the hosts for various purposes would have more specific requirements. For example, when used as a cell factory for production of components or additives of cosmetics, medicines and foods, a host suitable for safe, green and industrializable bio-manufacturing would be required.

Cyanobacteria are a group of oxygen-evolving photosynthetic prokaryotes that utilize the light as the energy and CO_2_ as the carbon source, and are major participants in the geochemical cycles of carbon, nitrogen and oxygen (Hamilton et al., 2016; Lobus and Kulikovskiy, 2023). There is a great interest in engineering cyanobacteria as hosts for photosynthetic conversion of CO_2_ into high value-added products. In recent years, some laboratory model strains, for instance, *Synechococcus elongatus* PCC 7942, *Synechococcus* sp. PCC 7002 and *Synechocystis* sp. PCC 6803, have been utilized as hosts for the production of biofuels, polymers, pigments and many other value-added chemicals, such as isoprene (Gao et al., 2016), ethanol (Kopka et al., 2017), PHB (Koch et al., 2020), astaxanthin (Diao et al., 2020), limonene (Lin et al., 2017), squalene (Choi et al., 2017), amino acids (Korosh et al., 2017; Brey et al., 2020), fatty acids (Włodarczyk et al., 2020). These efforts have demonstrated the enormous potential of cyanobacteria as model organisms for carbon-negative synthetic biology (Tan et al., 2022). However, these strains have been limited in commercial applications, due to slow growth (relative to heterotrophic bacteria), poor resistance to adversity, predation by protozoa, etc. Therefore, the development of more robust and industrializable strains to serve as cyanobacteria model organisms is critical for future commercial processes.

There are some cyanobacterial strains with superior growth traits meeting the requirements of commercial production. For example, *Arthrospira* (*Spirulina*) *platensis* is a filamentous cyanobacterium with the characteristics of high safety, high protein content, fast growth, convenient harvest, and excellent environmental adaptability, thus is commercially farmed worldwide as a food source (Ahmad et al., 2023). It has long been hoped to be developed as a host platform for syntheses of proteins and chemicals. A recent report showed that exogenous genes cloned on plasmids were transformed into this cyanobacterium with the aid of companion bacteria, integrated into the chromosome via homologous double crossover and efficiently expressed (Jester et al., 2022). However, the described genetic transformation was not based on colony formation on plates, and the segregation process appeared to be very time consuming. Such a technical bottleneck may limit the development of more sophisticated genetic systems for synthetic biology. An alternative filamentous cyanobacterium suitable for large-scale cultivation, called *Leptolyngbya* sp. BL0902 (hereafter *Leptolyngbya* BL0902), was initially isolated from an algal production raceway pond (Taton et al., 2012; Ma et al., 2014). It exhibits a series of superior traits, including fast growth in a wide temperature range (22°C ~ 40°C) and high tolerance to salt, alkalinity and light stresses, and is amenable to conjugal gene transfer (based on colony formation on plates) (Taton et al., 2012).

So far, genetic alterations of *Leptolyngbya* BL0902 depended on the expression of exogenous genes on RSF1010-derived plasmids (Taton et al., 2012; Ma et al., 2014; Poole et al., 2020). To be established as a model strain, its genome sequence and gene annotation must be published, and efficient genetic manipulations of large/small regions on the chromosome should be demonstrated. In this study, we analyzed the genome sequence of *Leptolyngbya* BL0902 and performed different types of genetic manipulations with the existing genetic tools and strategies. Our results indicated that this strain could be developed into an excellent model strain for metabolic engineering and synthetic biology studies in cyanobacteria.

## 2 Materials and methods

### 2.1 Strains, growth conditions and conjugation

*Leptolyngbya* BL0902 was from Dr. Golden JW (University of California-San Diego). *Leptolyngbya* BL0902 and derivatives were grown in BG11 in flasks, with manual agitation 3–4 times a day, at 30°C under the illumination of 30 μE·m^−2^·s^−1^. For selection with antibiotics, spectinomycin (10 μg/mL) or neomycin (25 μg/mL) was added to liquid or solid media as appropriate.

The growth of *Leptolyngbya* BL0902 and a 50.8 kb-deletion mutant in Zarrouk medium (3 biological replicates) was compared in vertical column-type photobioreactors (48.5 cm × 2.0 cm) bubbled with air supplemented with 1% CO_2_ in the light of 100 μE·m^−2^·s^−1^. The OD_730_ value was adjusted to 0.05 at the beginning and measured every 12 h.

Conjugation was performed as described by Taton et al. (2012), using *Escherichia coli* HB101 containing pRL443 (conjugative plasmid), pRL623 (helper plasmid) and the plasmid to be transferred into *Leptolyngbya* BL0902 as the donor strain. The helper plasmid is not required for the conjugal transfer of RSF1010-based editing plasmids, but it is not necessary to remove this plasmid from the donor strain.

### 2.2 Semi-continuous cultivation in a raceway photobioreactor

**Figure 1:**
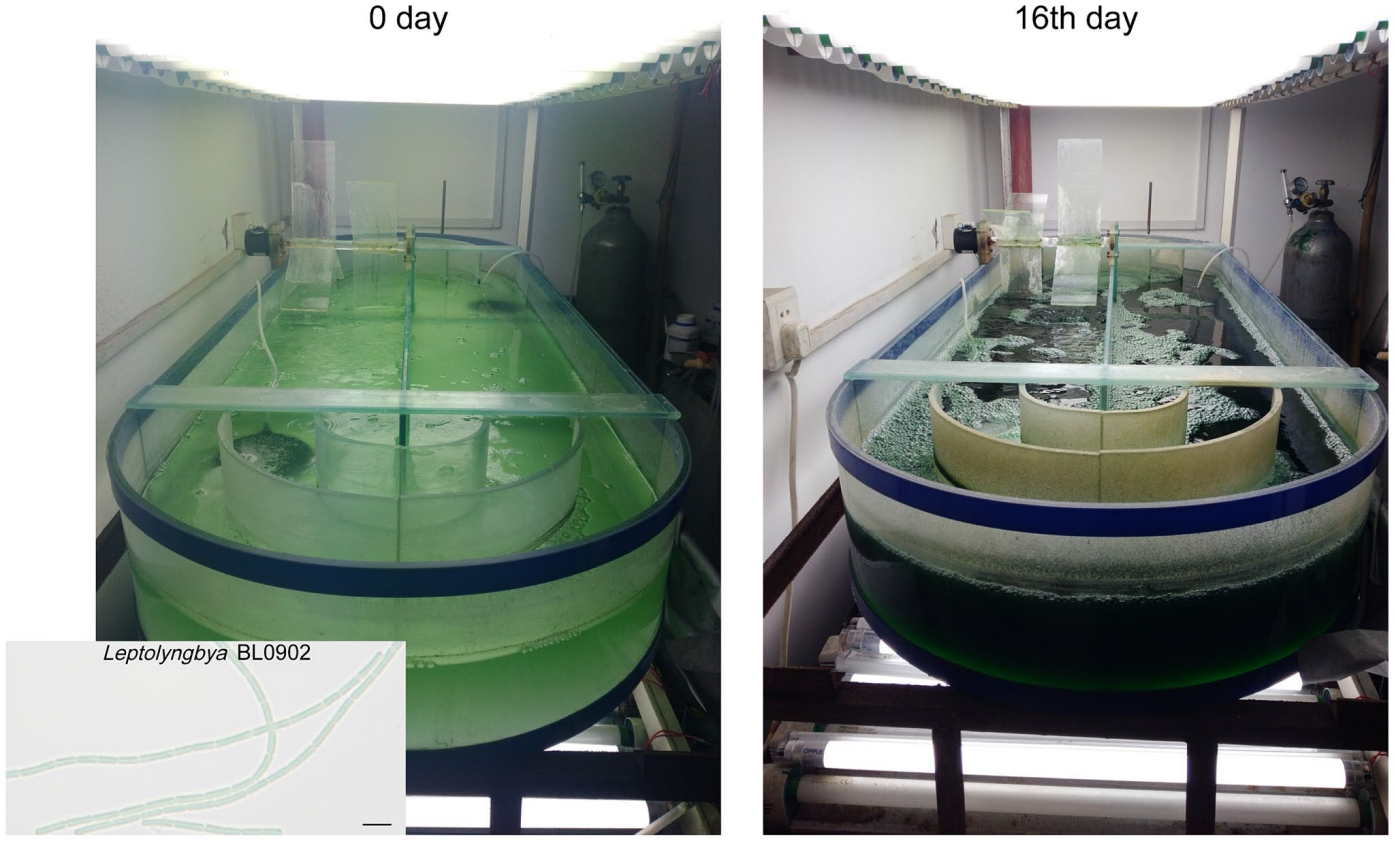
Cultivation of *Leptolyngbya* BL0902 P*_psbA_*-*desD* in the open raceway photobioreactor. The scale bar in the photomicrograph at the lower left corner stands for 5 μm.

The 100 L-scale semi-continuous cultivation of *Leptolyngbya* BL0902 P*_psbA_*-*desD* was carried out in Zarrouk medium bubbled with 4% CO_2_ (v/v) at 26°C ~ 29°C, in a 1-m^2^ open raceway photobioreactor, with the constant illumination of 100 μE·m^−2^·s^−1^ from both upper and lower sides (Figure 1). The depth of the culture was kept at 10 cm by replenishing water every day to compensate for the evaporative loss. An electric motor-driven paddle wheel, 0.3 m in diameter, rotating at 20 rpm, was used to propel the circulating movement of the culture. The initial OD_730nm_ of the culture was ~0.1. Starting from the 5th day after the inoculation, cells were harvested once every 2 days; at each harvest time point, 50 L of the culture were collected, and the same volume of fresh medium was supplemented to the culture. The growth of cells was recorded based on the dry weight of biomass per liter in two biological replicates. At each time point, 50 mL of cells taken from the culture were vacuum filtered, washed with 0.5 N HCl and dried at 105°C for 4 h, and the dry weight was measured.

### 2.3 Gas chromatography analyses of fatty acid composition and γ-linolenic acid (GLA) contents

Fatty acid composition was analyzed as previously described (Laurens et al., 2012) with 3 biological replicates. Lipids were extracted from 10 mg freeze-dried cells and simultaneously transesterified, with sequentially added chloroform/methanol (2:1) and 5% HCl in methanol, and analyzed by GC (Trace Ultra, Thermo Electric) equipped with a DB-23 capillary column (60 m in length, 0.25 mm in diameter, 0.25 μm in film thickness) coupled to a FID detector. For quantification of the GLA content in *Leptolyngbya* strains, tridecanoic acid (C13:0) was added to the dried cells as an internal standard before extraction and transesterification of lipids.

### 2.4 Construction of plasmids and *Leptolyngbya* mutants

Molecular cloning manipulations were performed according to standard protocols, but some ligation reactions were performed using ClonExpress Ultra One step Cloning Kit V2 (Vazyme, Nanjing, China) based on *in vitro* homologous recombination. DNA fragments generated by PCR were confirmed by sequencing after cloning in plasmids. Details of plasmid construction and strain generation are provided in Supplementary Table S1 but briefly described as below.

For interruption of *desB* with the *omega* cassette (Prentki and Krisch, 1984) or P*_psbA_*-*desD*-*omega* in *Leptolyngbya* BL0902, pHB6115 and pHB6127 were constructed, with these fragments inserted at the Bal I site of *Leptolyngbya desB*, cloned into pRL271, a *sacB*-bearing vector (Cai and Wolk, 1990). The *desD* gene was generated by PCR using *Synechocystis* PCC 6803 DNA as the template.

For generation of deletions in the chromosome of *Leptolyngbya* BL0902, a DNA fragment with the two homologous arms for double crossover recombination was generated by overlap PCR (Horton et al., 1989) and cloned into the Bgl II-BamH I site of a pCpf1 plasmid, and a short dsDNA with the gRNA sequence was cloned into the plasmid replacing the DNA fragment between the two Aar I sites. Such constructed plasmids pHB7912, pHB7913, pHB7914 and pJS2529 were used to generate the Δ*hetR*, Δ[*patX*-*hetR*], Δ*patX* and Δ07990-08580 mutants, respectively.

These plasmids were introduced into *Leptolyngbya* BL0902 by conjugation (Taton et al., 2012), and exconjugants were randomly picked and streaked on plates with appropriate antibiotics, followed by selection in liquid medium. Genomic DNA was extracted from these exconjugants with a quick mini-preparation method (Cai and Wolk, 1990) for PCR examinations.

### 2.5 Genome sequencing and annotation

High-molecular-weight genomic DNA was extracted from *Leptolyngbya* BL0902 using the cetyltrimethylammonium bromide (CTAB) method (Murray and Thompson, 1980), and RNA was removed by using RNase A. The genome sequence was determined by third generation long read sequencing technology on PacBio platforms (Pacific Biosciences, CA, United States). A total of 283,232 reads were obtained, encompassing 1.7 Gb, with an average read length of 5,990 bp. In addition, Illumina sequencing was performed on Illumina HiSeq 2,500 (Illumina Inc., San Diego, CA, USA) to obtain high quality reads, producing 3 Gb high quality data. The genome was assembled using HGAP3 (Chin et al., 2013) based on PacBio sequencing reads and polished using Illumina reads. Finally, the *Leptolyngbya* BL0902 genome was assembled into a complete circular chromosome with no gap and five circular plasmids.

Protein-coding genes were predicted by using Glimmer3 (Delcher et al., 2007) with default parameters, and the predicted proteins were annotated by searching against NCBI non-redundant protein (NR),^1^ Swiss-Prot (Wu et al., 2006), COG (Tatusov et al., 1997), KEGG (Kanehisa and Goto, 2000) and GO (Ashburner et al., 2000) databases. The rRNA sequences were identified using RNAmmer (Lagesen et al., 2007), tRNA genes identified using tRNAscan-SE (Lowe and Eddy, 1997), tandem repeats (> 6 bp) identified using Trf407b.linux in RepeatMasker,^2^ and regularly interspaced short palindromic repeat (CRISPR) arrays identified using MinCED.^3^

### 2.6 Transcriptomic analyses

*Leptolyngbya* strains grown to OD_730nm_ ~ 0.8 were collected by centrifugation, quickly frozen and stored in liquid nitrogen. Total RNA was extracted from cells using the RNA pre-purification Cell/Bacteria Kit (Tiangen Biotech Co., Ltd., Beijing, China), then DNA was removed with DNase I, and rRNA was removed using the Ribo-off rRNA Depletion Kit V2 (Bacteria) (Vazyme, Nanjing, China). One microgram of total RNA was used to construct RNA libraries after retro-transcription, and 150 nt paired-end sequencing was performed on Illumina Novaseq 6,000.

The raw paired-end reads were trimmed and quality controlled by fastp version 0.21.0 (Chen et al., 2018) with default parameters, then clean reads (over 3 G for each strain) were separately aligned to reference genome using hisat2 version: 2.0.1-beta (Kim et al., 2015). The featureCounts function from the Subread package (Liao et al., 2013) was used to count reads that mapped to each one of the protein-coding genes. Raw count data was then used as input into DESeq2 v.1.42.0 (Love et al., 2014) for differential expression analyses (3 biological replicates). Differential expression was considered significant if the absolute FoldChange value was >2 or < 0.5 and the false discovery rate (FDR) adjusted *p*-value was <0.05. After applying a regularized-logarithm transformation to the raw count gene matrix, we calculated the average expression value of each gene across three biological replicates, then standardized the expression of each gene along the samples to generate a standardized matrix and visualized the result as a heatmap using the package ggplot2 v.3.4.4 (Wickham, 2016), in R.

## 3 Results

### 3.1 Analyses of the genome sequence of *Leptolyngbya* BL0902

The genome of *Leptolyngbya* BL0902 (GenBank accession numbers: CP046155-CP046160) is composed of six circular DNA molecules, the chromosome and five plasmids (Table 1), with a total size of 4.71 Mb. The 4.309 Mb-long chromosome is predicted to have 3,981 protein-coding genes, 6 rRNA (two copies of rRNA operons) and 43 tRNA genes; the five plasmids, with sizes of 158.483 kb, 93.510 kb, 78.547 kb, 44.466 kb and 25.969 kb, contain 177, 84, 66, 68 and 27 protein-coding genes, respectively. To our best knowledge, this is the smallest among the *Leptolyngbya* genomes (Table 2) and those of the closely related genus *Nodosilinea* (Supplementary Table S2). As seen in some cyanobacteria with rRNA operon variants, such as *Anabaena* sp. PCC7120 (Iteman et al., 2000), the two 16S-23S internal transcribed spacer regions of *Leptolyngbya* BL0902 contain either tRNA-Ile or tRNA-Ala gene. In addition, 571 tandem repeats (> 6 bp) and 16 clustered regularly interspaced short palindromic repeat (CRISPR) arrays were identified in the genome (Table 1).

**Table 1:** General information about the genome of *Leptolyngbya* BL0902.

Sequence	Length (bp)	Number of protein-coding genes	Number of rRNAs	Number of tRNAs	Number of tandem repeats	Number of CRISPR arrays
Chromosome	4,309,234	3,981	6	43	537	13
Plasmid 1	158,483	177	–	–	11	2
Plasmid 2	93,510	84	–	–	11	–
Plasmid 3	78,547	66	–	–	8	–
Plasmid 4	44,466	68	–	–	3	1
Plasmid 5	25,969	27	–	–	1	–
Total	4,710,209	4,403	6	43	571	16

**Table 2:** Comparison of *Leptolyngbya* genomes.

Organism	Genome size (bp)	Number of protein-coding genes	Type II restriction endonuclease	*nif* * cluster	NCBI GenBank accession no.
*Leptolyngbya* sp. BL0902	4,710,209	4,403	No	No	CP046155.1-CP046160.1
*Leptolyngbya boryana* dg5	6,803,469	6,295	No	Yes	NZ_AP014642-NZ_AP014645
*Leptolyngbya ohadii* IS1	7,902,459	7,487	Yes	Yes	NZ_NKFP00000000
*Leptolyngbya* sp. NIES-2104	6,386,310	6,712	Yes	No	NZ_BBWW00000000
*Leptolyngbya boryana* PCC 6306	7,262,454	6,715	No	Yes	NZ_KB731324-NZ_KB731328
*Leptolyngbya* sp. PCC 7376	5,125,950	4,525	No	No	NC_019683
*Leptolyngbya* sp. O-77	5,480,261	4,865	Yes	Yes	NZ_AP017367
*Leptolyngbya* sp. NIES-3755	6,761,657	6,521	Yes	No	NZ_AP017308-NZ_AP017311
*Leptolyngbya boryana* NIES-2135	7,233,668	6,674	No	Yes	NZ_AP018203-NZ_AP018206
*Leptolyngbya* sp. PCC 7375	9,422,068	8,102	No	Yes	NZ_JH993793-NZ_JH993797
*Leptolyngbya* sp. PCC 6406	5,769,257	5,080	Yes	Yes	NZ_KI913949-NZ_KI913951
*Leptolyngbya* sp. KIOST-1	6,320,123	5,663	Yes	Yes	NZ_JQFA00000000
*Leptolyngbya* sp. ‘hensonii’	5,940,030	5,233	Yes	Yes	MQTZ00000000

*Nitrogen fixation genes.

Some cyanobacterial species produce cyanotoxins that pose risks to human and animals, and the syntheses of cyanotoxins are typically dependent upon conserved gene clusters. We searched the genome of *Leptolyngbya* BL0902 for all the eleven representative cyanotoxin biosynthesis gene clusters (Pearson et al., 2016) but found no similar one. Type II restriction enzymes may cut foreign DNA and greatly reduce gene transfer efficiency in cyanobacteria (Elhai et al., 1997). However, no genes (homologs) for type II restriction enzymes from REBASE (Roberts et al., 2010) were found in the genome of *Leptolyngbya* BL0902.

Cyanobacteria can be classified into 4 groups according to the types of fatty acid desaturases (Murata and Wada, 1995): (1) those only with DesC that generates a single double bond at position 9 (Δ9, from the C-terminus) on the fatty acid chain in acyl-lipids; (2) those with DesA (Δ12), DesB (Δ15) and DesC; (3) those with DesA, DesC and DesD (Δ6); (4) those with DesA, DesB, DesC and DesD. In the genome of *Leptolyngbya* BL0902, we found genes encoding DesA (GFS31_05880), DesB (GFS31_39000) and DesC (GFS31_27570), therefore it should be a strain of group 2.

In filamentous cyanobacteria, there is a monophyletic group that form specialized cells termed heterocysts for nitrogen fixation (Wolk et al., 1994). Some non-heterocystous cyanobacteria can also perform nitrogen fixation (Rippka and Waterbury, 1977), and these diazotrophic cyanobacteria all possess the *nif* (nitrogen fixation) gene cluster (Tsujimoto et al., 2014). *Leptolyngbya* BL0902 is a non-heterocystous cyanobacterium without the *nif* gene cluster; however, it possesses genes (GFS31_32400, GFS31_16620-GFS31_16630) similar to *hetR* (Buikema and Haselkorn, 2001) and *hetZ*-*patU* (Zhang et al., 2007), which play central roles in regulation of heterocyst differentiation. In heterocyst-forming cyanobacteria, HetR directly regulates the expression of *hetZ* via a HetR-binding site, while HetZ regulates the expression of *hetR* and two genes encoding RG(S/T)GR-containing peptides, namely *patS* and *patX*, directly or indirectly via the DIF1 promoter (Du et al., 2020). HetR also activates or inhibits the expression of some other genes, such as *hetP* and the *hetP*-like gene *alr3234* in the heterocyst-forming cyanobacterium *Anabaena* sp. PCC 7120 (Hou et al., 2015). The pentapeptide RG(S/T)GR, derived from PatS (Yoon and Golden, 1998), PatX (Elhai and Khudyakov, 2018) and a protein called HetN (Higa et al., 2012), is an inhibitor of HetR. In *Leptolyngbya* BL0902, *patS* and *hetN* are not found, but a protein-encoding gene, GFS31_32390, meets the definition of *patX* (Elhai and Khudyakov, 2018); of the three ORFs similar to *hetR*, the one (GFS31_32400) with the highest similarity is located immediately downstream of *patX* and appears to be co-transcribed with *patX* from the predicted DIF1 promoter (Supplementary Figure S1).

### 3.2 Generation of a stable GLA-producing strain by replacing *desB* with P*_psbA_*-*desD*

**Figure 2:**
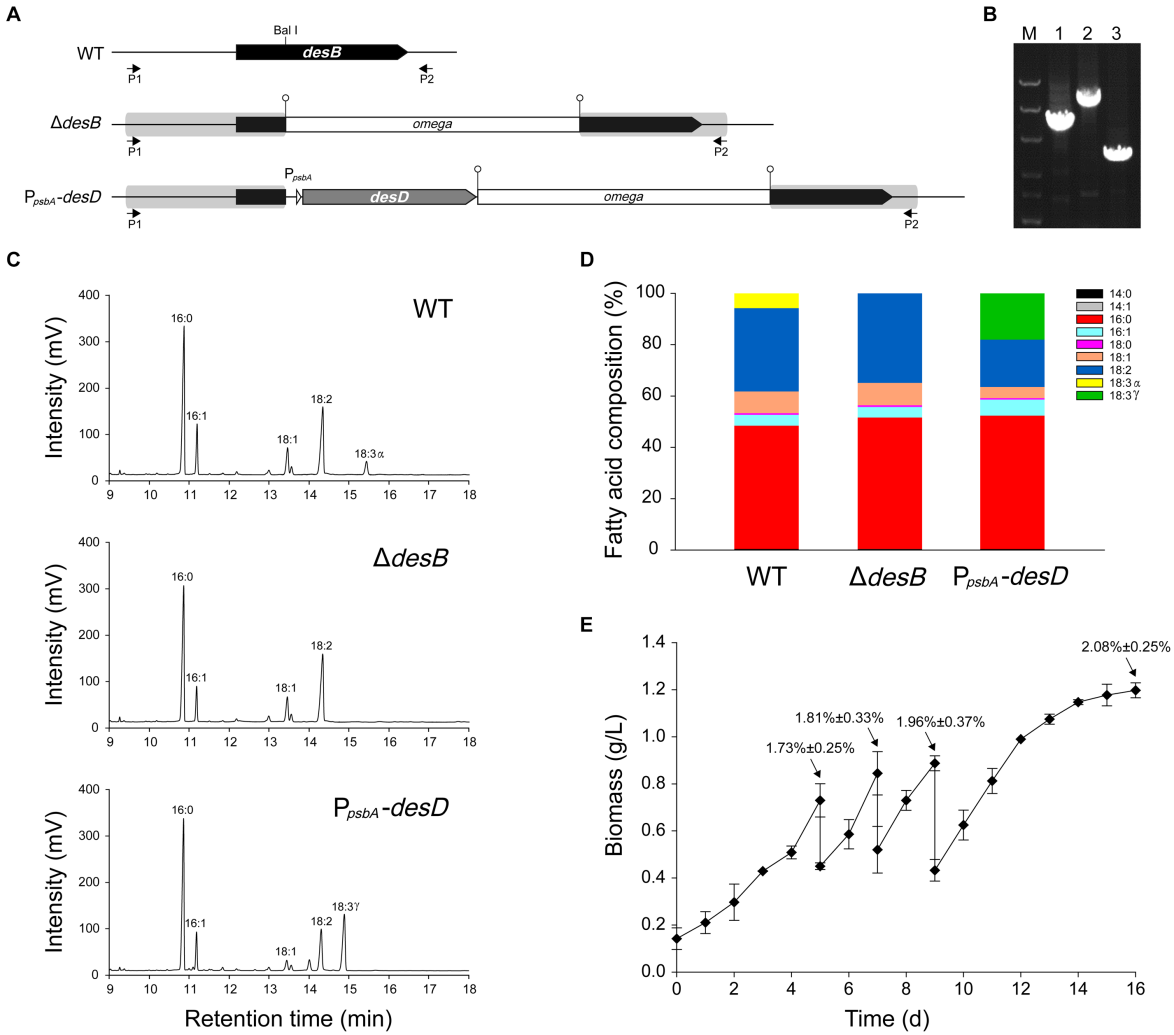
Generation of a *Leptolyngbya* BL0902 strain that produces GLA as the only C18:3 fatty acid. **(A)** The structure of the *desB* region in the wild type (WT) and two mutant strains Δ*desB* and P*_psbA_-desD*. P*_psbA_-desD* is the GLA-producing strain. P1 and P2 represent the primers, L-desB-1 and L-desB-2, for PCR examination. **(B)** PCR examination of the *desB* region, as shown in the electrophoretogram: lane 1, △*desB*; lane 2, P*_psbA_-desD*; lane 3, WT. M, dsDNA marker (8 kb, 5 kb, 3 kb, 1.5 kb, 1 kb, 0.5 kb). **(C)** Gas chromatograms of fatty acid methyl esters prepared from acyl-lipids of *Leptolyngbya* strains. **(D)** Fatty acid compositions of *Leptolyngbya* strains (also see Supplementary Table S3). **(E)** Growth of the P*_psbA_-desD* strain under semi-continuous cultivation conditions in the open raceway photobioreactor (see Figure 1). Percentages (mean ± SD) indicated at the four time points are GLA contents of cells (w/w).

A gene transfer system based on wide-host-range plasmids has been established in *Leptolyngbya* BL0902 (Taton et al., 2012; Poole et al., 2020). However, manipulations of genetic loci on the chromosome are required for gene function analyses and generation of more stable cell factories. To this end, we tested integration of genes into the chromosome by homologous double-crossover. In *Leptolyngbya* BL0902, the three fatty acid desaturases generate double bonds at Δ9, Δ12 and Δ15 positions on C_18_ fatty acid chains of acyl-lipids, leading to the formation of α linolenic acid (ALA). First, we constructed the plasmid pHB6125 (Supplementary Table S1) for interrupting *desB* with the *omega* cassette (Sp^r^) in *Leptolyngbya* BL0902 (Figures 2A,B). A DNA fragment containing *desB* was cloned in the plasmid, and the *omega* cassette was inserted at the Bal I site of *desB*. *sacB* on the vector can provide the positive selection for double-crossover recombinants. We planned to introduce the plasmid into *Leptolyngbya* BL0902 by conjugation to obtain single-crossover recombinants, then to select double-crossover mutants on sucrose-containing plates. However, when we checked 9 randomly picked spectinomycin-resistant exconjugants by PCR examinations, one of them was already double-crossover mutant (Δ*desD*), whereas the rest 8 were single-crossovers, therefore the selection on sucrose-containing plates was omitted. Then, we tried to interrupt *desB* with P*_psbA_*-*desD* and the *omega* cassette, so as to replace α-linolenic acid with γ-linolenic acid (GLA, C_18_ fatty acid with double bonds at Δ6, Δ9 and Δ12) in *Leptolyngbya* BL0902. P*_psbA_* is a strong promoter from the chloroplast of *Amaranthus hybridus* (Elhai, 1993), while *desD* is from *Synechocystis* PCC 6803. The plasmid pHB6127 was constructed in a structure similar to pHB6125, but a fragment with P*_psbA_*-*desD* and the *omega* cassette was inserted into the BalI site of *desB*. Of 11 randomly picked exconjugants, 10 formed single-crossover recombination between pHB6127 and the chromosome, but one directly formed double-crossover, with P*_psbA_*-*desD* and the *omega* cassette inserted within *desB*, and this *desD*-overexpressing strain was called P*_psbA_*-*desD* (Figures 2A,B).

Gas chromatography analyses of fatty acid composition showed that unlike in the wild type, ALA was no longer formed in the Δ*desB* mutant (Figures 2C,D; Supplementary Table S3). In the P*_psbA_*-*desD* strain, ALA (18:3α) was replaced with GLA (18:3γ), which accounted for 18.02% ± 0.92% of long chain fatty acids. Compared to the fatty acid composition in the wild type, C18:3 increased in this strain at the expense of mono- and di-unsaturated C18 fatty acids (Figure 2D; Supplementary Table S3). The *desD*-overexpressing strain and GLA content were stable over past 6 years under laboratory conditions. To demonstrate the potential for commercial production, we evaluated the biomass and GLA productivities of this strain under semi-continuous cultivation conditions in a 1-m^2^ open raceway photobioreactor (Figures 1, 2E). On the 5th, 7th and 9th day, half of the culture was collected for cell harvest, and fresh medium of the same volume was supplemented to the culture. Productivities were calculated based on the increase from the 5th day to the 7th day and that from the 7th day to the 9th day (3 samples × 2). The cultivation was extended to the 16th day. The GLA contents gradually increased at the four time points, reaching ~2% of the biomass (dry weight). Based on two independent cultivation experiments (3 samples × 2 × 2), we calculated that the productivity of biomass was 19.1 ± 4.9 g·m^−2^·d^−1^, and that of GLA was 0.36 ± 0.14 g·m^−2^·d^−1^ (*p* < 0.05).

### 3.3 Markerless deletion of *patX*-*hetR* and transcriptomic analyses

**Figure 3:**
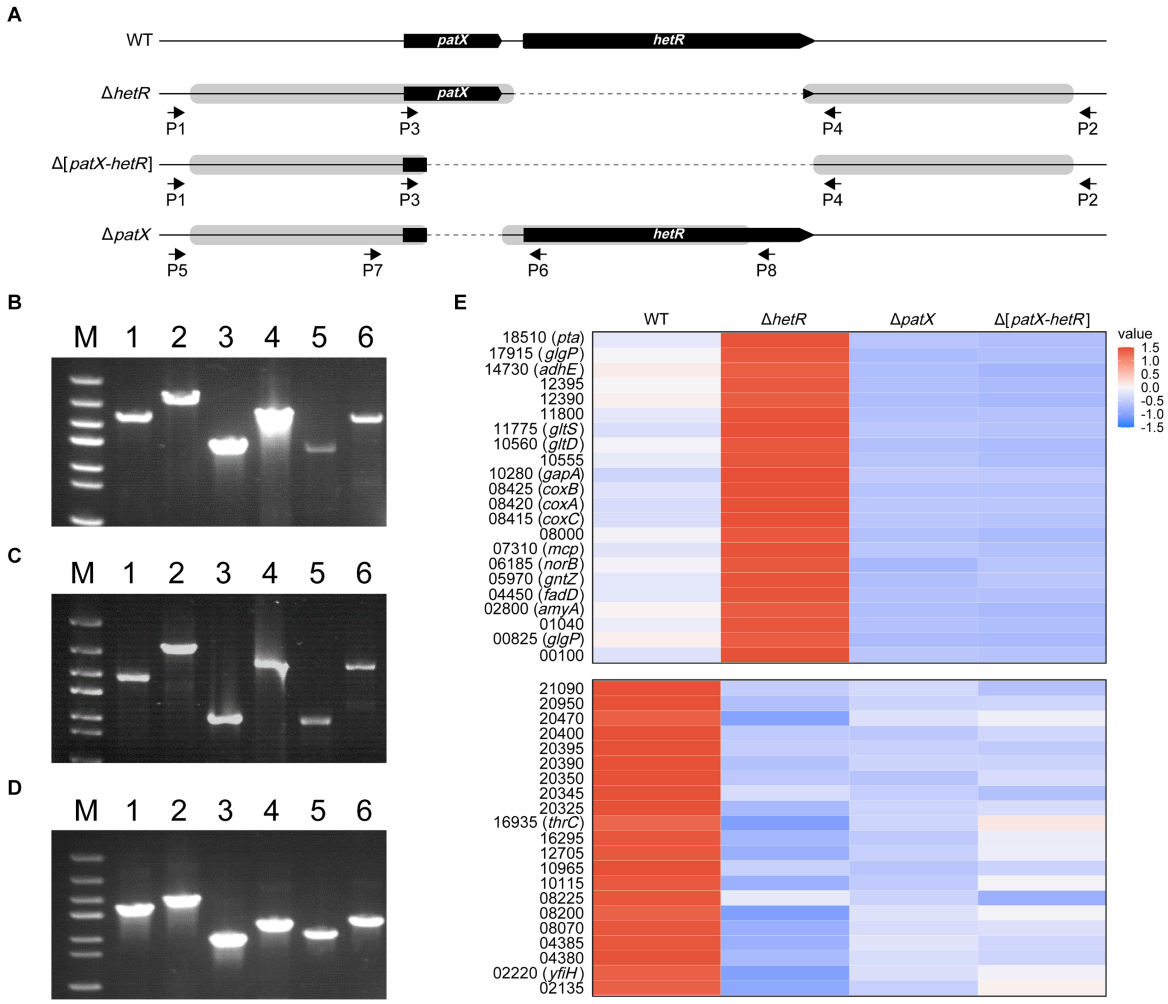
Markerless deletions at the *patX*-*hetR* region of *Leptolyngbya* BL0902. **(A)** The structure of the *patX*-*hetR* region in the WT and mutant strains Δ*hetR*, Δ[*patX-hetR*] and Δ*patX*. P1, P2, P3, P4, P5, P6, P7 and P8 represent the primers Ch-0902-hetR-F, Ch-0902-hetR-R, Ch-patX-hetR-F-in, Ch-hetR-R-in, Ch-patX-F1, Ch-patX-rev, Ch-patX-For and Ch-patX-R1, respectively. **(B–D)** Electrophoretograms of PCR products for examination of the mutants (lanes 1, 3, 5), with the wild type as the control (lanes 2, 4, 6). Primers used in B and C (Δ*hetR*, Δ[*patX-hetR*]): lanes 1 and 2, P1/P2; lanes 3 and 4, P1/P4; lanes 5 and 6, P3/P2. Primers used in D (Δ*patX*): lanes 1 and 2, P5/P8; lanes 3 and 4, P5/P6; lanes 5 and 6, P7/P8. M, dsDNA marker (5 kb, 3 kb, 2 kb, 1.5 kb, 1 kb, 0.8 kb, 0.5 kb). **(E)** A partial heatmap showing two types of differential expression patterns in the three mutants and the wild type. The full heatmap is shown in Supplementary Figure S2, based on the standardized expression levels of the genes identified in RNA-seq analyses (Supplementary Table S4). The blue shades designate decreasing levels, and red shades increasing levels. The color intensity denotes the standardized expression, as indicated by the scale bar.

For analyses of gene functions or biotechnological genetic manipulations, markerless deletions or insertions are sometimes required. The CRISPR/Cpf1 editing system is suitable for such purposes (Ungerer and Pakrasi, 2016; Niu et al., 2018). *hetR* and *patX* are two genes found in almost all filamentous cyanobacteria, heterocyst-forming or not (Elhai and Khudyakov, 2018). The functions of these two genes in those species that do not form heterocysts remain a mystery. We constructed Cpf1-based editing plasmids pHB7912, pHB7913 and pHB7914 (Supplementary Table S1), for deleting *hetR*, *patX*-*hetR* and *patX*, respectively. The editing plasmids were introduced into *Leptolyngbya* BL0902 by conjugation, and exconjugants were checked with PCR using specific primers (Figures 3A–D; Supplementary Table S1). In the resulted mutants Δ*hetR*, Δ[*patX*-*hetR*] and Δ*patX*, the predicted DIF1 promoter upstream of *patX*-*hetR* (Supplementary Figure S1) remained unchanged. The editing plasmids were then removed from the mutants by positive selection on sucrose-containing plates, and the removal of plasmids was confirmed by PCR examination and antibiotic-resistance assay (data not shown).

In heterocyst-forming cyanobacteria, PatX is thought to be one of the precursors for the RG(S/T)GT-containing inhibitor of HetR (Elhai and Khudyakov, 2018; Khudyakov et al., 2020). We wondered whether PatX counteracts the regulatory effects of HetR, either activating or inhibitory, on gene expression in *Leptolyngbya* BL0902. Transcriptomic analyses were performed to compare the transcriptional profiles of the WT and mutants. The results showed that there were 993 genes differentially expressed in Δ*hetR vs* the wild type, 428 in Δ*patX*, 477 in Δ[*patX*-*hetR*], including genes involved in photosynthesis, respiration, nitrogen metabolism, etc. Of these genes, 126 were up- or down-regulated in all these mutants compared to the wild type. According to the differential expression patterns in mutants compared to the wild type, these 126 genes can be classified into 5 groups (Supplementary Figure S2, with 2 groups shown in Figure 3E as examples). It appeared that HetR downregulates genes in the first and second groups but upregulates those in the third, fourth and fifth groups. Only in groups 2 and 3, PatX showed opposite effects on gene expression compared to HetR. If PatX acts as a (pre)inhibitor of HetR, the effect of *patX* on gene expression should be dependent upon that of *hetR*. However, in groups 2 and 3, Δ[*patX*-*hetR*] was similar to Δ*patX* but contrary to Δ*hetR* in differential expression. Apparently, the effects of PatX on gene expression are basically independent of HetR in *Leptolyngbya* BL0902.

### 3.4 Markerless deletion of a large nonessential region in the chromosome

**Figure 4:**
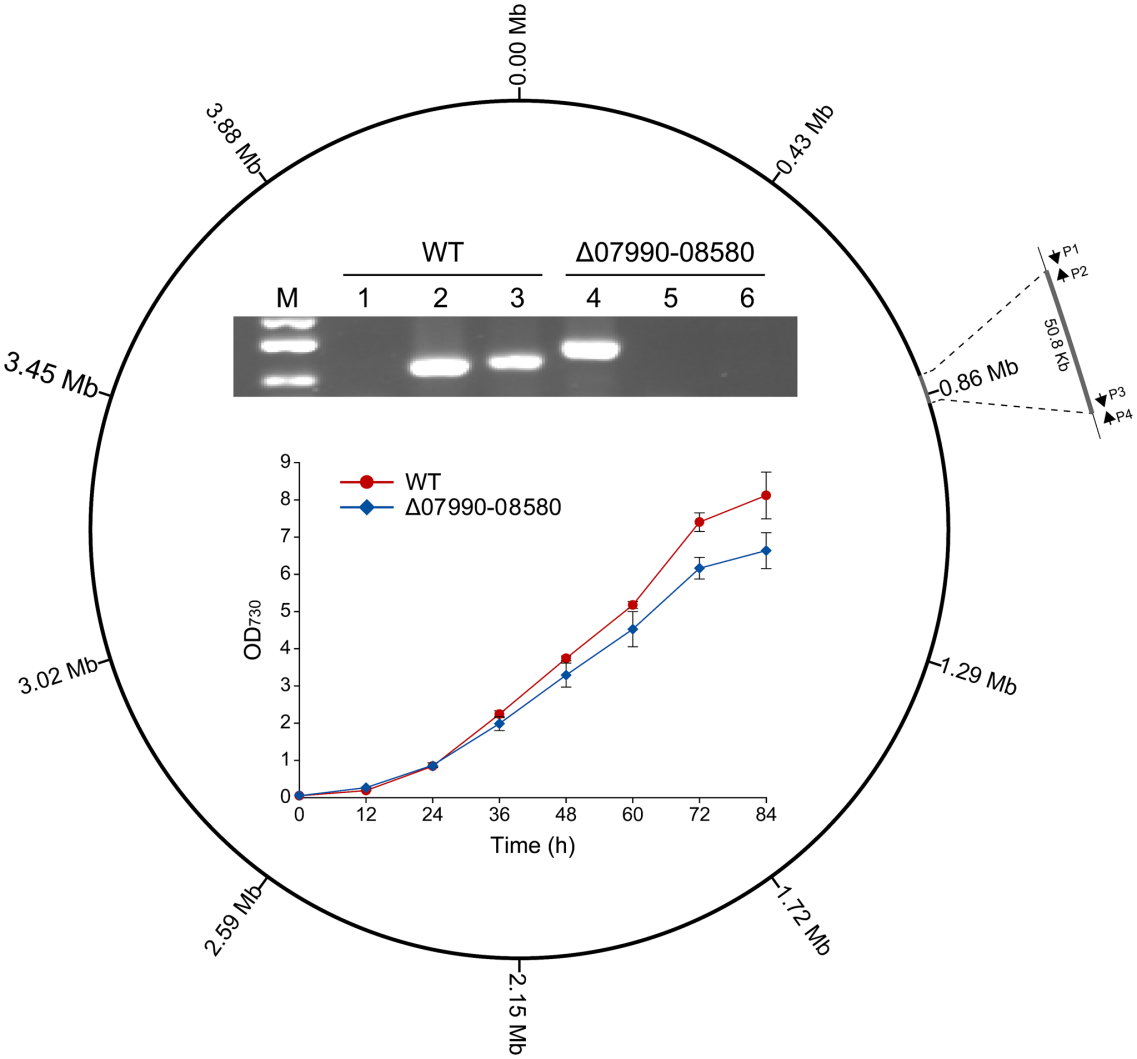
Deletion of a 50.8-kb region in the chromosome of *Leptolyngbya* BL0902, generating the Δ07990-08580 mutant. P1, P2, P3 and P4 represent the primers BL0783-0842-F, BL0783-0842-R′, BL0783-0842-F′ and BL0783-0842-R. The electrophoretogram shows the result of PCR examination of the mutant: lanes 1 and 4, using primers P1/P4; lanes 2 and 5, P1/P2; lanes 3 and 6, P3/P4. M, dsDNA marker (0.75 kb, 0.5 kb and 0.25 kb). The growth of the mutant and the wild type was compared in Zarrouk medium in column-type photobioreactors.

Deletions of large non-essential regions in the chromosome are required for genome streamlining in cyanobacteria, which would reduce the genomic complexity and may improve some productive traits (Hou et al., 2023; Sengupta et al., 2024). By BLAST searching against the essential genes experimentally identified in *Synechococcus elongatus* PCC 7942 (Rubin et al., 2015), we obtained a list of genes that are probably essential in *Leptolyngbya* BL0902. Then we tried to delete some chromosomal regions without these genes, for example, a 50.8-kb region (chromosomal bp 825,418–876,218), extending from GFS31_07990 to GFS31_08580. The Cpf1-based editing plasmid pJS2529 (Supplementary Table S1) was constructed and transferred into *Leptolyngbya* BL0902 by conjugation. Of 20 randomly picked exconjugants, 6 exhibited complete deletion of the 50.8-kb region. The complete segregation of the resulted mutant, Δ07990-08580, was confirmed by PCR using 3 pairs of primers (Figure 4). When grown in Zarrouk medium in column photobioreactors with aeration, Δ07990-08580 showed a slightly reduced growth rate compared to that of the wild type (Figure 4), but this does not compromise the feasibility of genome streamlining in *Leptolyngbya* BL0902.

## 4 Discussion

For biological research in cyanobacteria, there are some frequently used model strains, such as unicellular species *Synechocystis* sp. PCC 6803, *Synechococcus* sp. PCC 7942 and *Synechococcus* sp. PCC 7002, and heterocyst-forming filamentous species *Anabaena*/*Nostoc* sp. PCC 7120 and *A.* var*iabilis* ATCC 29413. However, no filamentous cyanobacteria that do not form heterocysts have been widely used as a model strain. This is probably due to two reasons: (1) lack of a focus question that needs to be addressed with this type of cyanobacteria; (2) lack of a strain that is suitable for different types of genetic manipulations. However, with the development of synthetic biology in cyanobacteria, it is increasingly realized that a non-heterocystous filamentous model strain is necessary, because a filamentous strain, if it is tolerant to a high concentration of sodium bicarbonate and grows quickly (for example, in Zarrouk medium), is more suitable for commercial-scale cultivation and low-cost harvest than unicellular species. On the other hand, for the sake of basic research, to find out how heterocysts originated or how genes involved in heterocyst differentiation originated, our efforts must be extended to filamentous species that do not form heterocysts. *Leptolyngbya* BL0902 is such a filamentous cyanobacterium, with the potential to be developed into a model strain. In the whole genome sequence of this strain, we found no genes involved in cyanotoxin biosynthesis and no genes encoding type II restriction enzymes. Therefore, it could be a safe host for production of nutrients, food/cosmetics additives or constituents of medicine, and the existing genetic tools and strategies may be directly used in manipulations of its genomic DNA.

Genetic manipulations in filamentous cyanobacteria usually depend on conjugal transfer of plasmids from *E. coli* to recipient cells (Elhai et al., 1997) and single/double-crossover recombination between the homologous sequence(s) on the plasmid and the target genomic DNA. Double-crossover mutants are often generated employing positive selection strategies, such as the use of a *sacB* gene on a non-replicable vector (Cai and Wolk, 1990) or a CRISPR/Cpf1 system on a replicative plasmid (Ungerer and Pakrasi, 2016). The former strategy is expected to generate the single-crossover mutant at the first step, then from the single cross-over mutant, double crossovers are selected based on the lethal effect of *sacB* on cyanobacterial cells grown on sucrose-containing plates. However, in *Leptolyngbya* BL0902, we found that about 1/10 of exconjugants generated at the first step were already double-crossover mutants, therefore the second step was omitted. The mechanism for the high ratio of double-crossover mutants in the exconjugants remains to be elucidated. One possibility is that a substantial proportion of the plasmid was linearized after transfer into *Leptolyngbya* cells. Apparently, this strategy is only suitable for insertion of a DNA fragment with an antibiotic-resistance marker into the target sequence; for generation of markerless deletions in the chromosome of *Leptolyngbya* BL0902, the CRISPR/Cpf1 system would be much more efficient.

Based on the genetic manipulation system, we generated a *Leptolyngbya* strain that produced GLA as the only C18:3 fatty acid and four markerless deletion strains. The GLA-producing strain was semi-continuously cultivated with an open raceway photobioreactor, and the result demonstrated that value-added chemicals could be stably produced in *Leptolyngbya* BL0902 in a way potentially for commercial production (Figures 1, 2). Of the four deletion mutants, three were used to analyze the relationship of *hetR* and *patX* in non-heterocystous filamentous cyanobacterium. Although PatX contributes to the inhibition of HetR in *Anabaena* PCC 7120, the transcriptomic analysis of the differential expression between Δ*hetR*, Δ[*patX*-*hetR*], Δ*patX* mutants and the wild type of *Leptolyngbya* BL0902 did not show a similar effect of PatX on HetR (Figure 3). Probably, PatX acts as an independent regulator in *Leptolyngbya*. In addition to deletions in the *patX*-*hetR* region, we also generated a mutant in which a 50.8-kb nonessential region was deleted (Figure 4). Generation of the large fragment deletion in the chromosome further lends support to our proposal that *Leptolyngbya* BL0902 could be used as a model strain for synthetic biological studies in filamentous cyanobacteria. The affected growth of the large-fragment deletion mutant (or multiple-deletion mutants afterwards) may be restored by using a hypermutation system (Sun et al., 2023).

In the future, *Leptolyngbya* BL0902 may be further developed into ready-to-use ‘plug and play’ chassis cells. First, the genome can be extensively streamlined. In bacterial genomes, there are many nonessential regions that are disposable under favorable conditions (Martínez-García and de Lorenzo, 2016; Kurokawa and Ying, 2019). Elimination of these regions can lower genomic complexity and improve the predictability and operability of genetic engineering. Second, different types of regulatory modules may be integrated into the genome. Such gene expression platforms would allow desired products to be synthesized under specific conditions or at the stationary growth phase, so as to alleviate the contradiction between cell propagation and product accumulation. In addition to the applied purposes, gene function analyses in *Leptolyngbya* BL0902 may contribute to the studies on the origin of heterocysts, and reconstruction of functional modules of heterocyst differentiation in this strain would greatly consolidate research models proposed for heterocyst formation and patterning.

## Data availability statement

The *Leptolyngbya* sp. BL0902 genome sequences with annotations have been deposited in the NCBI GenBank under accession numbers CP046155.1 (chromosome), CP046156.1 (plasmid 1), CP046157.1 (plasmid 2), CP046158.1 (plasmid 3), CP046159.1 (plasmid 4) and CP046160.1 (plasmid 5). RNA-Seq raw sequence data are available at the NCBI BioProject repository under identification no. PRJNA1087288.

## Author contributions

HG: Funding acquisition, Investigation, Methodology, Writing – review & editing. YW: Funding acquisition, Investigation, Writing – original draft. ZH: Investigation, Writing – original draft. FY: Investigation, Writing – original draft. XH: Validation, Visualization, Writing – review & editing. DN: Funding acquisition, Supervision, Writing – original draft, Writing – review & editing. XX: Conceptualization, Funding acquisition, Project administration, Supervision, Writing – original draft, Writing – review & editing.

## Footnotes

^1^ www.ncbi.nlm.nih.gov

^2^ http://www.repeatmasker.org

^3^ https://github.com/ctSkennerton/minced/tree/master
