# Supplementary material for: Development of Leptolyngbya sp. BL0902 into a model organism for synthetic biological research in filamentous cyanobacteria
Source: Front Microbiol. 2024 Jul 22;15:1409771. doi: 10.3389/fmicb.2024.1409771 (PMC11298460; doi:10.3389/fmicb.2024.1409771)
Supplement: Supplementary file 1 [file Table_1.doc]

**Table S1. Cyanobacterial strains, p**lasmids and primers

| ***Leptolyngbya* BL0902 strains** | **Derivation and relevant characteristics** | **Reference or source** |
| --- | --- | --- |
| WT | The wild type strain isolated from an open raceway cultivation pond. | Taton et al, 2012 |
| △*desB* | Sp^r^, a *desB*::*omega* mutant, constructed by homologous double crossover between pHB6115 and the chromosome in the WT strain. | This study |
| P*_psbA_-desD* | Sp^r^, a *desB*::P*_psbA_*-*desD*-*omega* mutant, constructed by homologous double crossover between pHB6127 and the chromosome in the WT. |
| Δ*hetR* | A mutant with *hetR* deleted, constructed by CRISPR/Cpf1-based positive selection of the deletion, mediated by double-crossover recombination between the editing plasmid pHB7912 and the chromosome in the WT, followed by removal of the plasmid. |
| Δ*patX* | A mutant with *patX* deleted, constructed by CRISPR/Cpf1-based positive selection of the deletion, mediated by double-crossover recombination between the editing plasmid pHB7914 and the chromosome in the WT, followed by removal of the plasmid. |
| Δ[*patX*-*hetR*] | A mutant with both *patX* and *hetR* deleted, constructed by CRISPR/Cpf1-based positive selection of the deletion, mediated by double-crossover recombination between the editing plasmid pHB7913 and the chromosome in the WT, followed by removal of the plasmid. |
| Δ07990-08580 | A mutant with a 50.8-kb chromosomal region extending from GFS31_07990 to GFS31_08580 deleted, constructed by CRISPR/Cpf1-based positive selection of the deletion, mediated by double-crossover recombination between the editing plasmid pJS2529 and the chromosome in the WT, followed by removal of the plasmid. |
|  | | |
| **Plasmids** | **Derivation and relevant characteristics*^a^*** | **Reference or source** |
| pMD18-T | Ap^r^, T-vector | TaKaRa |
| pRL57 | Km^r^Sm^r^Sp^r^, a pDU1-based plasmid with the *omega* cassette containing the streptomycin/spectinomycin-resistance gene | Elhai and Wolk, 1988 |
| pRL271 | Cm^r^(Em^r^), a *sacB*-bearing cloning vector with *oriV* and *oriT* sites from pMB1 | Black et al, 1993 |
| pRL439 | Ap^r^, a plasmid carrying P*_psbA_* promoter | Elhai, 1993 |
| pRL443 | Ap^r^Tc^r^, a Km-sensitive derivative of the conjugal plasmid RP4 | Elhai et al, 1997 |
| pRL623 | Cm^r^, the helper plasmid with ColK replicon, the *mob* gene from ColK, and genes encoding M.AvaI, M.Eco47II and M.EcoT22I |
| pCpf1-Sp | Sp^r^, a CRISPR-Cpf1-based editing vector | Niu et al, 2018 |
| pCpf1-Km | Km^r^/Nm^r^, a CRISPR-Cpf1-based editing vector |
| pHB6104 | Ap^r^, a 2017-bp DNA fragment containing the *desB* gene, generated by PCR using primers L-desB-1 and L-desB-2, cloned into pMD18-T | This study |
| pHB6105 | Ap^r^, the DNA fragment with *desD* from *Synechocystis* sp. PCC 6803, generated by PCR using primers desD-1 and desD-2, cloned into pMD18-T |
| pHB6111 | Sp^r^, the *omega* cassette excised from pRL57 with Dra I, cloned into PstI-cut and T4 DNA polymerase-blunted pHB6105, generating *desD*-*omega* |
| pHB6114 | Sp^r^, the *omega* cassette excised from pRL57 with Dra I, cloned into BalI-digested pHB6104, generating *desB*::*omega* |
| pHB6115 | Sp^r^, *desB*::*omega* excised from pHB6114 with Spe I, ligated to SpeI-cut pRL271 |
| pHB6119 | Sp^r^, *desD*-*omega* excised from pHB6111 with Pvu II and BamH I, cloned into EcoR I and BamH I-cut and T4 DNA polymerase-blunted pRL439，generating P*_psbA_-desD*-*omega* |
| pHB6125 | Sp^r^, P*_psbA_-desD*-*omega* excised from pHB6119 with Pst I and Hind III, blunted with T4 DNA polymerase, cloned into Bal I-cut pHB6104, generating *desB*::P*_psbA_-desD*-*omega* |
| pHB6127 | Sp^r^, *desB*::P*_psbA_-desD*-*omega* excised from pHB6125 with Spe I , cloned into Spe I-cut pRL271 |
| pHB7890 | Km^r^/Nm^r^, a DNA fragment with two homologous arms for deletion of *patX*, including a 769-bp region overlapping the 5’ end of *patX* and a 805-bp region downstream of *patX*, generated by overlap PCR using primers 0902-patX-1/0902-patX-2 and 0902-patX-3/ 0902-patX-4, ligated to Bgl II and BamH I-cut pCpf1-Km via seamless cloning |
| pHB7908 | Km^r^/Nm^r^, a DNA fragment with two homologous arms for deletion of *hetR*, including a 1051-bp region upstream of *hetR* and a 927-bp region overlapping the 3’ end of *hetR*, generated by overlap PCR using primers 0902-hetR-1/0902-hetR-2 and 0902-hetR-3/0902-hetR-4, ligated to Bgl II and BamH I-cut pCpf1-Km via seamless cloning |
| pHB7909 | Km^r^/Nm^r^, a DNA fragment with two homologous arms for deletion of *patX*-*hetR*, including a 769-bp region overlapping the 5’ end of *patX* and a 842-bp region downstream of *hetR*, generated by overlap PCR using primers 0902-hetR-1/0902-patX-hetR-2 and 0902-patX-hetR-3/0902-hetR-4, ligated to BglII-BamHI-cut pCpf1-Km via seamless cloning |
| pHB7912 | Km^r^/Nm^r^, the short dsDNA generated by annealing Sg-hetR-1 and Sg-hetR-2, cloned into Aar I-cut pHB7908 as the gRNA sequence |
| pHB7913 | Km^r^/Nm^r^, the short dsDNA generated by annealing sg-hetR-5 and sg-hetR-6, cloned into Aar I-cut pHB7909 as the gRNA sequence |
| pHB7914 | Km^r^/Nm^r^, the short dsDNA generated by annealing sg-patX-1 and sg-patX-2, cloned into Aar I-cut pHB7890 as the gRNA sequence |
| pJS2331 | Sp^r^, the short dsDNA generated by annealing g0842-F and g0842-R, cloned into Aar I-cut pCpf1-Sp as the gRNA sequence |
| pJS2529 | Sp^r^, a DNA fragment with two homologous arms for deletion of chromosomal bp 825418-876218, including a 800-bp region upstream of GFS31_07990 and a 803-bp region overlapping the 3’ end of GFS31_08580, generated by overlap PCR using primers BL0783-0842-F1/BL0783-0842-R1 and BL0783-0842-F2/ BL0783-0842-R2, ligated into BamH I and Bgl II-cut pCpf1-Sp by seamless cloning |
|  | | |
| **Primers** | **Sequences (5′→3′)** | |
| 0902-hetR-1 | tggcagaaattcgatatctaCCCAGTAGGGCGCAGGGATGATCACCTCATCGCC | |
| 0902-hetR-2 | TGGTACTTATCGGCCCAGGCCCGAATCCCACACGCAGTGTAGCCTGTTCTGGGC | |
| 0902-hetR-3 | GCCCAGAACAGGCTACACTGCGTGTGGGATTCGGGCCTGGGCCGATAAGTACCA | |
| 0902-hetR-4 | gcaacgttgttgccattgcgCGCCATCAAAGCCGCCAAGTCTCGCCAGGAGCCCT | |
| 0902-patX-1 | tggcagaaattcgatatcta CCCAGTAGGGCGCAGGGATGATCACCTCATCGCC | |
| 0902-patX-2 | GCCTGTTCTGGGCTGGGTAGGATAGCGGGAGGGCTACGCCTAGGGTAAAACCCGC | |
| 0902-patX-3 | GCGGGTTTTACCCTAGGCGTAGCCCTC CCGCTATCCTACCCAGCCCAGAACAGGC | |
| 0902-patX-4 | gcaacgttgttgccattgcgGTATCCTCAATCATGGCGTACATGCGT | |
| 0902-patX-hetR-2 | TCTGTGGGGTTTGGGGGGCTAACCCGAGGGCTACGCCTAGGGTAAAACCCGC | |
| 0902-patX-hetR-3 | GCGGGTTTTACCCTAGGCGTAGCCCTCGGGTTAGCCCCCCAAACCCCACAGA | |
| BL0783-0842-F | GTCGGTTCCTGGATAAGGTG | |
| BL0783-0842-F' | GTCGAGGTGCTTCAGGTAGG | |
| BL0783-0842-F1 | tggcagaaattcgatatctaTAGCGGCCTTCTAGAAGCCG | |
| BL0783-0842-F2 | GGAGGTTCGATGGCTTGCACAAGATCACCGCATTCTGGTCAGG | |
| BL0783-0842-R | TGGCGCAGAAATTCCAGCAC | |
| BL0783-0842-R' | CCTGCCGACGCAGGTGCTCC | |
| BL0783-0842-R1 | TTGTGCAAGCCATCGAACCTCC |  |
| BL0783-0842-R2 | gcaacgttgttgccattgcgTGCATTCGGTCAATGGTGCG | |
| Ch-0902-hetR-F | GCCGCAGTTGATCGGGGGTAATGCGA | |
| Ch-0902-hetR-R | GCAGTCGGCCCTAAAGGAAGTGCTC | |
| Ch-hetR-R-in | AGGGATCGACCGAAGCCGACGGTC | |
| Ch-patX-F1 | CCGCCAACTGCACCATCTCAGGA | |
| Ch-patX-For | AGTCACGATCCGCGTCACAATCCGCG | |
| Ch-patX-hetR-F-in | CGCTTGGCCCGTTCGCTGCTGCT | |
| Ch-patX-R1 | TGGCCCATTCGCGCATCATGCGG | |
| Ch-patX-rev | TTGATCGATGGCGCTGGGGCTGAG | |
| desD-1 | gcggatccCTGCTAAGGAGGCAACAAGATGCTAACAGCGGAAAGAATTA | |
| desD-2 | TTTGCTTCAATCCCAAGGCA | |
| g0842-F | agatTGGACGCTCGATAACCACACCT | |
| g0842-R | agacAGGTGTGGTTATCGAGCGTCCA | |
| L-desB-1 | gcactagtCCGCCATGATCAAAGTAAGCCACGG | |
| L-desB-2 | gcactagtATCAGGGGTAGACAGCCAGGAT | |
| Sg-hetR-5 | agatAAGGATCTCGATGAAATGATTC | |
| Sg-hetR-6 | agacGAATCATTTCATCGAGATCCTT | |
| sg-patX-1 | agatGTGATTCTCCGTTGACGGCAGG | |
| sg-patX-2 | agacCCTGCCGTCAACGGAGAATCAC | |

Km, kanamycin; Nm, neomycin; Sp, spectinomycin.

*^a^* Unless stated otherwise, the template for PCR reactions was *Leptolyngbya* genomic DNA.

**References**

Black TA, Cai Y, Wolk CP (1993) Spatial expression and autoregulation of*hetR*, a gene involved in the control of heterocyst development in *Anabaena*. Mol Microbiol 9: 77-84.

Elhai J, Wolk CP (1988) A versatile class of positive-selection vectors basted on the nonviability of palindrome-containing plasmids that allows the cloning into long polylinkers. Gene 68: 119-138.

Elhai J (1993) Strong and regulated promoters in the cyanobacterium *Anabaena* PCC 7120. FEMS Microbiol Lett. 114: 179-184.

Elhai J, Vepritskiy A, Muro-Pastor AM, Flores E, Wolk CP (1997) [Reduction of conjugal transfer efficiency by three restriction activities of *Anabaena* sp. strain PCC 7120.](https://pubmed.ncbi.nlm.nih.gov/9068647/) J Bacteriol 179: 1998-2005.

Niu TC , Lin GM, Xie LR, Wang ZQ, Wei-Yue Xing WY, Zhang JY ,  Zhang CC (2018) Expanding the potential of CRISPR-Cpf1-based genome editing technology in the cyanobacterium *Anabaena* PCC 7120. ACS Synth Biol. 8: 170-180

Taton A, Lis E, Adin DM, Dong G, Cookson S, Kay SA, Golden SS, Golden JW (2012) Gene transfer in *Leptolyngbya* sp. strain BL0902, a cyanobacterium suitable for production of biomass and bioproducts. PLoS One. 7: e30901.
