# Supplementary material for: Development of Leptolyngbya sp. BL0902 into a model organism for synthetic biological research in filamentous cyanobacteria
Source: Front Microbiol. 2024 Jul 22;15:1409771. doi: 10.3389/fmicb.2024.1409771 (PMC11298460; doi:10.3389/fmicb.2024.1409771)
Supplement: Supplementary file 2 [file Table_2.DOCX]

**Table S2 Genome sizes of *Nodosilinea* strains**

| Organism | Genome size (Mb) | Number of protein-coding genes | NCBI GenBank accession no. |
| --- | --- | --- | --- |
| *Nodosilinea nodulosa* PCC 7104 | 6.9 | 6098 | GCF_000309385.1 |
| *Nodosilinea* sp. E11 | 6.1 | 5140 | GCF_032813545.1 |
| *Nodosilinea* sp. FACHB-13 | 5.7 | 4802 | GCF_014696165.1 |
| *Nodosilinea* sp. FACHB-141 | 5.9 | 5092 | GCF_014696135.1 |
| *Nodosilinea* sp. TSF1-S3 | 6.4 | 5475 | GCF_029109325.1 |
| *Nodosilinea* sp. LEGE 06152 | 6.3 | 5409 | GCF_015207525.1 |
| *Nodosilinea* sp. FACHB-131 | 6.2 | 5280 | GCF_014695395.1 |
| *Nodosilinea* sp. P-1105 | 6.0 | 5053 | GCF_012911975.1 |
| *Nodosilinea* sp. LEGE 07088 | 6.7 | 6073 | GCF_015207395.1 |
| *Nodosilinea* sp. LEGE 07298 | 7.3 | 6468 | GCF_015207265.1 |
| *Nodosilinea* sp. WJT8-NPBG4 | 5.9 | 5182 | GCA_019359285.1 |
