# Supplementary material for: Development of Leptolyngbya sp. BL0902 into a model organism for synthetic biological research in filamentous cyanobacteria
Source: Front Microbiol. 2024 Jul 22;15:1409771. doi: 10.3389/fmicb.2024.1409771 (PMC11298460; doi:10.3389/fmicb.2024.1409771)
Supplement: Supplementary file 3 [file Table_3.DOC]

**Table S3 Fatty acid composition (mol %) of the total glycerol lipids from *Leptolyngbya* BL0902 strains**

| Strains | C14:0 | C14:1 | C16:0 | C16:1 | C18:0 | C18:1 | C18:2 | C18:3γ (GLA) | C18:3α (ALA) |
| --- | --- | --- | --- | --- | --- | --- | --- | --- | --- |
| WT | 0.41±0.01 | 0.01±0.01 | 48.02±0.62 | 4.23±3.63 | 0.66±0.08 | 8.48±0.58 | 32.43±2.07 | 0.00 | 5.77±0.41 |
| ∆*desB* | 0.40±0.07 | 0.02±0.02 | 51.21±0.83 | 4.14±0.05 | 0.67±0.10 | 8.72±0.14 | 34.83±0.67 | 0.00 | 0.00 |
| P*_psbA_*-*desD* | 0.39±0.08 | 0.00±0.01 | 51.95±2.42 | 6.29±0.88 | 0.60±0.11 | 4.34±0.42 | 18.40±1.22 | 18.02±0.92 | 0.00 |
