# Supplementary material for: Development of Leptolyngbya sp. BL0902 into a model organism for synthetic biological research in filamentous cyanobacteria
Source: Front Microbiol. 2024 Jul 22;15:1409771. doi: 10.3389/fmicb.2024.1409771 (PMC11298460; doi:10.3389/fmicb.2024.1409771)
Supplement: Supplementary file 5 [file Data_Sheet_1.PDF]

```
1    GCCTGGGCATGGAGAGGGGGCACGTGGGGAGAGCCGAGATGGGCGACCCATCCTAGTCACGATCCGCGTCACAATCCGCG   80
81   AAGTTATGAAGGCAGAGTTTTCCTGACCGAGGTGATGGGGGGCTGTAACAAGGACTAGACGCGCTAAAAAGTCAATTCAC   160
161  TATGCAATCTTTCTCCGCTTGGCCCGTTCGCTGCTGCTTGGTCGGTGTTGCGGGTTTTACCCTAGGCGTAGCCCTCGTGG   240
241  TTAGTGATTCTCCGTTGACGGCAGGCCAGTCCGCTGAGCCTCGCTCATTTTTTCTAGAAGCCCAGGGCGAGGATCGGGGC   320
321  AGTGGTCGCCTCCATCAGCCCGCCTTGATCCATCAGGCCGCCATCCGGGGCAGTGGCCGCATCAGTCCTACCCCCCCGAC   400
401  ACCCGATGTTCAAGCCCAGACCACGGCCTACCGGGGCAGTGGTCGTATTGATCCCCACAGCCCCAACCGAGTCTAGCCGC   480
481  TATCCTACCCAGCCCAGAACAGGCTACACTGCGTGTGGGTGCCTTCTTAAGATCTTGATTTTCCGTACCCATGGCTGATT   560
561  CTGATCCGCAATCAACCGTTACATTTCCCGTTACCTACGATGAAGAACTGATTCGTCAGCTCAGCCCCAGCGCCATCGAT   640
641  CAAATTCTGCTGTATTTGGCCTTCAGTGCCATGCGGACTGGGGGGCACCGCCACGGGGCTTTTCTCGATGCGGCGGCCAC   720
721  GGCGGCCAAATGTGCCATCTACACCACCTACCTAGAGCAGGGCGAAAACCTGCGGATGACCGGGCATCTACACCACATCG   800
801  AGCCGAAGCGGGTGAAGGCGATTGTGGAGGAAATTCGACAGGCGCTGGTGGAGGGCAAGCTGCTGAAGCTGCTGGGTTCC   880
881  CAAGAGCCTCGCTACCTGATTCAGTTTCCCTATGTGTGGCTGCACCGCTACCCTTGGCAGGCCAAACAGTCGCGGGTTTT   960
961  AGGCACCTCCCTCACCCCCGATGAAAAGGCTAGTCTGGTCAGCAAGCTTCCCCCCCATAGCCCCCCAGCACGCATCATCA   1040
1041 ACTCCTTTCAGTTCCTAGAGCTGATTGAAGCCCTGCACGAAAAATCCCAAGAAGACTTGCCCTCCAGCCATCGCGTCGAT   1120
1121 CTCAGTGAAGCCCTCGCCGAACACATTCGCCGCCGCCTGATTTACTCCGGCACCGTGATTCGCCTGGATACCTATGGAGG   1200
1201 AGCCTCCTACTATGCCCTAGCGCGAAGTTCCTATTCCCCGGCTGACTCGGAAGAACGCATGTACGCCATGATTGAGGATA   1280
1281 CGGCCCAGTATTTCCGCATGATGCGCGAATGGGCCAGCCATCGTTCGGGAACCATGCGGGTGCTAGAAGAACTAGACATT   1360
1361 GCCGCTGAGGATGTGGATCGGGCCTTCAAGGATCTCGATGAAATGATTCGGGCCTGGGCCGATAAGTACCATCGTCCGGG   1440
1441 CGGCGAACCCATCCTGCTCCACATGGCCGTGGGCCACCATGCTCCCTAGGGGTTAGCCCCCCAAACCCCACAGAGCCTAG   1520
1521 ACGATCCGCATTTGACCGTCGGCTTCGGTCGATCCCTCGTCGTAGCCAAGGTGGCGGCGGGCGGCGGCG               1589
```

Figure S1 The sequence of the *patX-hetR* region in *Leptolyngbya* BL0902. The predicted DIF1 promoter is shaded in red (TCCTGA in the box resembles the TCCGGA motif), the encoding region of *patX* is in green, and *hetR* is in blue.

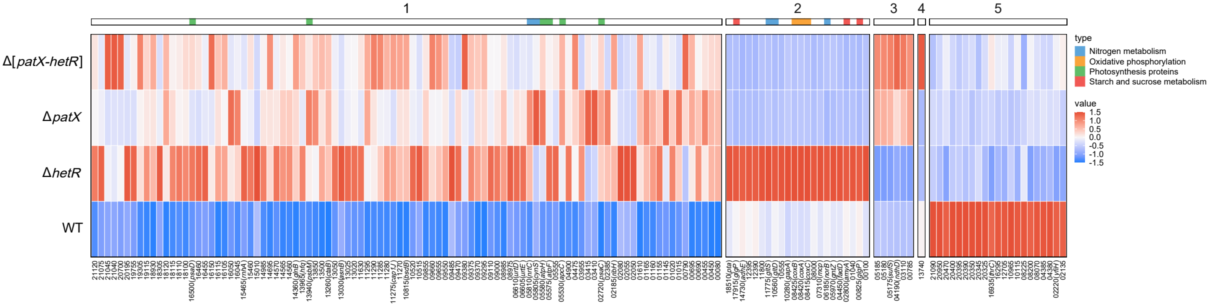

Figure S2 Heatmap showing the differential expression between the three mutants and the wild type. The 126 genes ('GFS31_RS' is omitted from the gene ID) are classified into 5 groups based on the differential expression pattern in the three mutants relative to the wild type.
